# Identification of the zinc finger 216 (ZNF216) in human carcinoma cells: a potential regulator of EGFR activity

**DOI:** 10.18632/oncotarget.12509

**Published:** 2016-10-06

**Authors:** Gabriella Mincione, Maria Carmela Di Marcantonio, Chiara Tarantelli, Luca Savino, Donatella Ponti, Marco Marchisio, Paola Lanuti, Silvia Sancilio, Antonella Calogero, Roberta Di Pietro, Raffaella Muraro

**Affiliations:** ^1^ Department of Medical, Oral and Biotechnological Sciences, University “G. d'Annunzio” Chieti-Pescara, Italy; ^2^ Center for Aging Science and Translational Medicine (CeSI-MeT), Chieti, Italy; ^3^ Department of Medico-Surgical Sciences and Biotechnologies, University of Rome Sapienza, Latina, Italy; ^4^ Department of Medicine and Ageing Sciences, University “G. d'Annunzio”, Chieti-Pescara, Italy; ^5^ Department of Pharmacy, University “G. d'Annunzio”, Chieti-Pescara, Italy; ^6^ Current Address: Lymphoma and Genomics Research Program, IOR Institute of Oncology Research, Bellinzona, Switzerland

**Keywords:** EGFR, ZNF216, nuclear EGFR

## Abstract

Epidermal Growth Factor Receptor (EGFR), a member of the ErbB family of receptor tyrosine kinase (RTK) proteins, is aberrantly expressed or deregulated in tumors and plays pivotal roles in cancer onset and metastatic progression. ZNF216 gene has been identified as one of Immediate Early Genes (IEGs) induced by RTKs. Overexpression of ZNF216 protein sensitizes 293 cell line to TNF-α induced apoptosis. However, ZNF216 overexpression has been reported in medulloblastomas and metastatic nasopharyngeal carcinomas. Thus, the role of this protein is still not clearly understood. In this study, the inverse correlation between EGFR and ZNF216 expression was confirmed in various human cancer cell lines differently expressing EGFR. EGF treatment of NIH3T3 cells overexpressing both EGFR and ZNF216 (NIH3T3-EGFR/ZNF216), induced a long lasting activation of EGFR in the cytosolic fraction and an accumulation of phosphorylated EGFR (pEGFR) more in the nuclear than in the cytosolic fraction compared to NIH3T3-EGFR cells. Moreover, EGF was able to stimulate an increased expression of ZNF216 in the cytosolic compartment and its nuclear translocation in a time-dependent manner in NIH3T3-EGFR/ZNF216. A similar trend was observed in A431 cells endogenously expressing the EGFR and transfected with *Znf216*. The increased levels of pEGFR and ZNF216 in the nuclear fraction of NIH3T3-EGFR/ZNF216 cells were paralleled by increased levels of phospho-MAPK and phospho-Akt. Surprisingly, EGF treatment of NIH3T3-EGFR/ZNF216 cells induced a significant increase of apoptosis thus indicating that ZNF216 could sensitize cells to EGF-induced apoptosis and suggesting that it may be involved in the regulation and effects of EGFR signaling.

## INTRODUCTION

Cellular signaling plays a very important role to regulate cell proliferation, survival and differentiation. The pathways involved in these cellular effects and the interactions among them are finely regulated with a high degree of specificity. Alterations of the cellular signaling could lead to pathology, as occurs in tumors. A very important role for the intracellular communication is played by cell-surface receptors which provide the interaction of cells with other cells and with the extracellular environment. One of the best characterized families of these cell-surface receptors is the ErbB family of receptor tyrosine kinases, that includes Epidermal Growth Factor Receptor (EGFR, ErbB1), ErbB2, ErbB3, and ErbB4. These receptors regulate a complex signaling network that impacts several cellular processes such as proliferation, survival, angiogenesis, and metastasis in many cancers.

EGFR is abnormally activated in many epithelial cells and its signaling can provide substantial advantage in tumor cells survival [1]. Abnormalities in gene expression, due to gene amplification, overexpression or mutations and alterations in signaling pathways downstream of the EGFR, contribute to the progression, invasion, and maintenance of the malignant phenotype in several human cancers, including head and neck, prostate, breast, bladder, ovarian, renal, colon, NSCLC [2]. All these findings support the role of EGFR as an important target for therapeutic intervention. Moreover, our group detected specific immune responses against all ErbB receptors in tumor patients with different epithelial malignancies including prostate cancer [3], and the presence of an autocrine loop in breast [4] and thyroid transformed cells [5].

The EGFR is activated by binding four different ligands, most commonly EGF and TGF-α. After ligand binding it forms homodimeric or heterodimeric complexes with other members of the ErbB family of receptors, preferably with ErbB2 [6].

Ligand binding and dimerization cause autophosphorylation of the intracytoplasmic domains and activation of the intracellular molecules such as the MAPK/ERK and the phosphoinositide-3 kinase (PI3K) pathways which regulate proliferation and cell survival [7]. After autophosphorylation causing its activation, EGFR is ubiquitinated and targeted for internalization [8, 9]. Once internalized, receptor complexes remain active, but eventually are either degraded within the lysosomes or recycled to the plasma membrane [10]. However, many studies demonstrated that EGFR could be shuttled from the plasma membrane to the nucleus after EGF stimulation [11] where it acts as a transcription co-factor [12–17]. In fact, interestingly, the EGFR lacks a DNA-binding domain and the nuclear EGFR physically interacts with other transcriptional molecules, such as the Signal Transducer and Activator of Transcription 3 (STAT3) to activate gene expression [14]. This interplay between EGFR activation, degradation and recycling, produces complex signaling layers interconnected by regulators with time and context restrictions (e.g., ligand type, gene transcription) that dictate EGFR-dependent responses (proliferation, survival, adhesion, differentiation, and migration) [18]. Thus, the different biological effects induced by activated EGFR and the regulation of its signaling may be due to its intracellular trafficking [19], to the expression of different genes, to modulation of individual pathways downstream of receptor activation and to its nuclear translocation [20, 21].

Our interest in zinc finger protein 216 (ZNF216) was attributable to its involvement in the interaction through its A20-like zinc finger domain with polyubiquitin chains and association with the 26S proteasome [22] and to the finding of its transcription as an immediate early gene (IEG) after activation of the tyrosine kinase receptors [23]. The ZNF216 expression is associated with atrophy in skeletal muscles, acting as a shuttle factor of ubiquitinylated proteins targeted to the 26S proteasome for degradation and the ZNF-A20 domain is required for binding to polyubiquitin [22]. These results support ZNF216's status as an atrogene. The A20 ZNF domain of ZNF216 and the UBA (Ub-associated domain) of the p62 protein are able to form an Ub-mediated ternary complex through independent interactions with a single Ub [24].

The ZNF216 gene was previously characterized during a study carried out to identify candidate genes for Autosomal Recessive Nonsyndromic Hearing Loss (ARNSHL) that are located in the DFNB7/11 interval in 9q13-q21 [25]. However, despite the position of ZNF216 on the human chromosome implicating a causal relationship to hearing loss, it could not be validated as a candidate. It has been reported that ZNF216 may inhibit TNF-induced NF-kB activation and sensitize cells to TNF-induced apoptosis [26]. In another study aimed at identifying differences in gene expression between medulloblastoma, a childhood brain tumor, and foetal brain, ZNF216 has been found expressed mostly in tumor tissue [27]. Moreover, ZNF216 has been found to be up-regulated in metastatic nasopharyngeal carcinoma cells compared to non-metastatic cells [28]. In addition, it has been shown that ZNF216 is among the 352 hepatic genes Keap-Nrf2-dependent induced by the D3T (3H-1,2-dithiole-3-thione), a detoxifying enzyme that enhances detoxication of environmental carcinogens and protect against neoplasia [29]. ZNF216 was found to be down-regulated together with EGFR in a mouse model of human EDA (Anhidrotic Ectodermal Dysplasia) by cDNA microarray hybridization experiments carried out to identify candidate genes for EDA-specific gene expression [30]. In this study in the attempt to verify whether ZNF216 could have a role in the EGFR signaling and in its biological outcomes, we set out to characterize the correlation between the EGFR and ZNF216 proteins in human cancer and to demonstrate whether a linkage between the EGFR-induced effects and ZNF216 expression/function does exist. Understanding the dynamics of this molecule and EGFR will be crucial in identifying potential new targets.

## RESULTS

### ZNF216 and EGFR expression in human cancer cell lines

ZNF216 is expressed in brain, hearth, colon, spleen, kidney, liver, small intestine, placenta, lung and peripheral blood leukocyte but highly expressed in skeletal muscle [25, 26, 31]. However, no information related to ZNF216 expression compared to EGFR expression in human cancer cell lines was available. A panel of five human tumor cell lines differently expressing EGFR was tested for ZNF216 and EGFR expression by RT-PCR and Western blotting.

**Figure 1:**
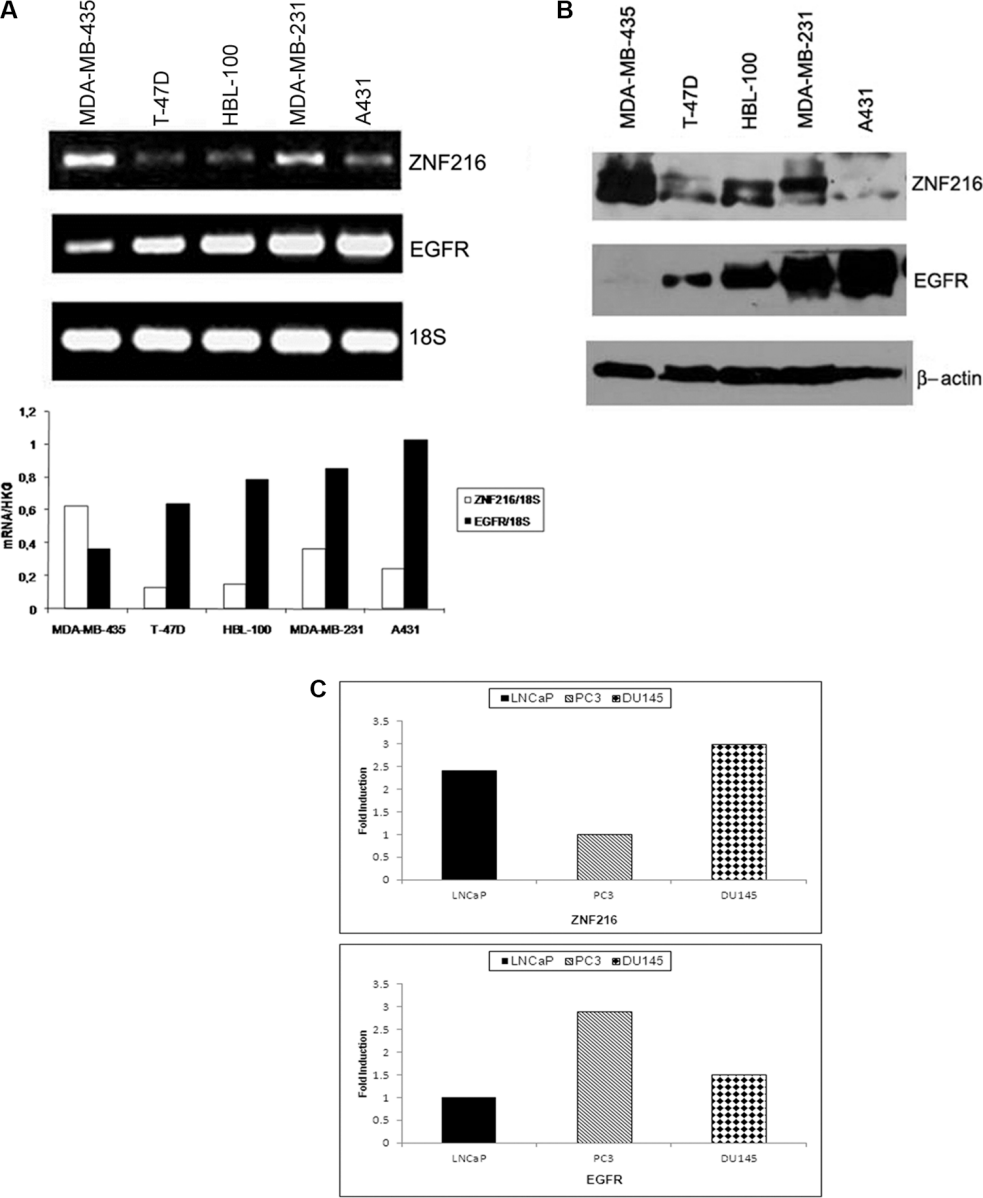
ZNF216 and EGFR expression in human cancer cell lines (**A**) ZNF216 and EGFR mRNA levels were detected in RNA samples from three human breast carcinoma (T-47D, HBL-100, MDA-MB-231), one human melanoma (MDA-MB-435) and one human epidermoid carcinoma (A431) cell lines, and analyzed by RT-PCR assay. The histogram describes the observed mRNA expression of ZNF216 and EGFR normalized with 18S in MDA-MB-435, T-47D, HBL-100, MDA-MB-231 and A431 cell lines. Results are representative of three independent experiments. (**B**) ZNF216 and EGFR protein levels were detected by Western blotting in MDA-MB-435, T-47D, HBL-100, MDA-MB-231 and A431 cell lines. β-actin was used as loading control. Results are representative of three independent experiments. (**C**) ZNF216 and EGFR mRNA relative expression levels were detected in RNA samples from LNCaP (androgen-dependent), PC3 and DU145 (androgen-independent) prostate cancer cell lines by quantitative Real Time PCR. The data are representative of three independent experiments.

RT-PCR analysis revealed that the ZNF216 mRNA was variably expressed among the cell lines (Figure 1A) with the highest level of expression found in melanoma cell line MDA-MB-435 weakly expressing EGFR and the lowest levels shown in breast carcinoma cell lines T-47D and HBL-100 moderately expressing EGFR mRNA [32] A431 cell line showed the highest level of EGFR gene expression, paralleled by a very low expression of ZNF216 mRNA. The ZNF216 protein levels detected on Western blotting (Figure 1B) were almost consistent with the pattern observed at the mRNA levels. Moreover, we selected three primary prostate cancer cell lines, LNCaP, PC3 and DU145, which are widely used and representative of advanced and highly tumorigenic prostate cancer to characterize the correlation between EGFR and ZNF216 expression through a Real-Time quantitative RT-PCR analysis [33–36]. LNCaP cell line, although isolated from a lymph node metastasis, represents a well differentiated and androgen responsive prostate carcinoma. As shown in Figure 1C DU145 cells showed the highest level of expression of ZNF216 mRNA. LNCaP cells expressed an intermediate level of ZNF216 mRNA while in PC3 cell line was detected the lowest expression level of ZNF216 transcript. On the other hand, PC3 cell line showed the highest level of EGFR expression compared to DU145 and LNCaP, confirming previously published results [37].

Thus, an inverse correlation between endogenous ZNF216 and EGFR level of expression was observed in these cell lines and is in accordance with those shown in prostate cancer cell lines.

### ZNF216 expression in NIH3T3 cells overexpressing ErbB receptors

To investigate the mechanism for a possible correlation between EGFR and ZNF216 expression we moved our attention on a model system consisting of fibroblast cell line stably expressing human EGFR, previously described (NIH3T3-EGFR) [38] and transfected it with a human ZNF216 cDNA (NIH3T3-EGFR/ZNF216).

**Figure 2:**
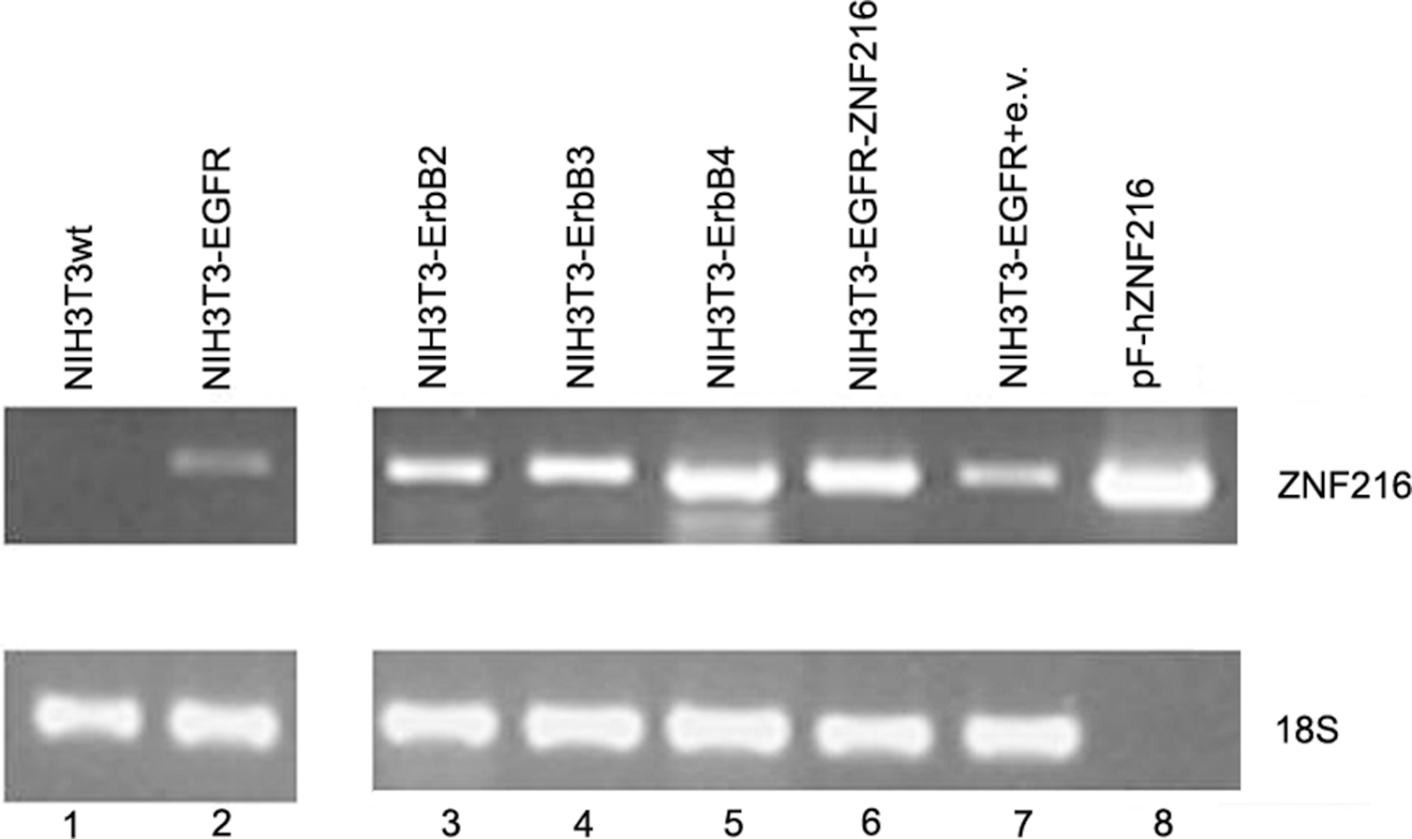
ZNF216 mRNA expression in NIH3T3 transfectant cell lines expressing individual ErbB receptors ZNF216 mRNA expression levels were detected in RNA extracted from NIH3T3 overexpressing individual ErbB receptors by RT-PCR assay. The expression level of 18S transcript was determined as a loading control. pF-hZNF216: expression vector containing the pF-hZNF216 cDNA (kindly provided by Dr. Watanabe). Data are representative of three independent experiments.

Total RNA was extracted from these cell lines and the expression of ZNF216 genes was evaluated through semiquantitative RT-PCR assay. The results of RT-PCR indicated that ZNF216 transcript was not detected in parental cell line NIH3T3wt and a low but detectable level of expression was seen in NIH3T3-EGFR (Figure 2, lanes 1 and 2). Moreover, in efforts to investigate whether other ErbB receptors, namely ErbB2, ErbB3 and ErbB4, were correlated to the expression of ZNF216 gene, we carried out an RT-PCR analysis in NIH3T3 overexpressing ErbB2, ErbB3 and ErbB4 genes in which the up-regulation of single ErbB receptor was validated when compared with NIH3T3 controls through Western blotting analysis (data not shown).

As shown in Figure 2, ZNF216 gene was expressed in all the cell lines analyzed with the strongest ZNF216 expression shown in NIH3T3-ErbB4 overexpressing cells (Figure 2, lane 5), thus suggesting that ErbB receptors could be involved in regulating ZNF216 expression.

### Ectopic expression of ZNF216 protein

**Figure 3:**
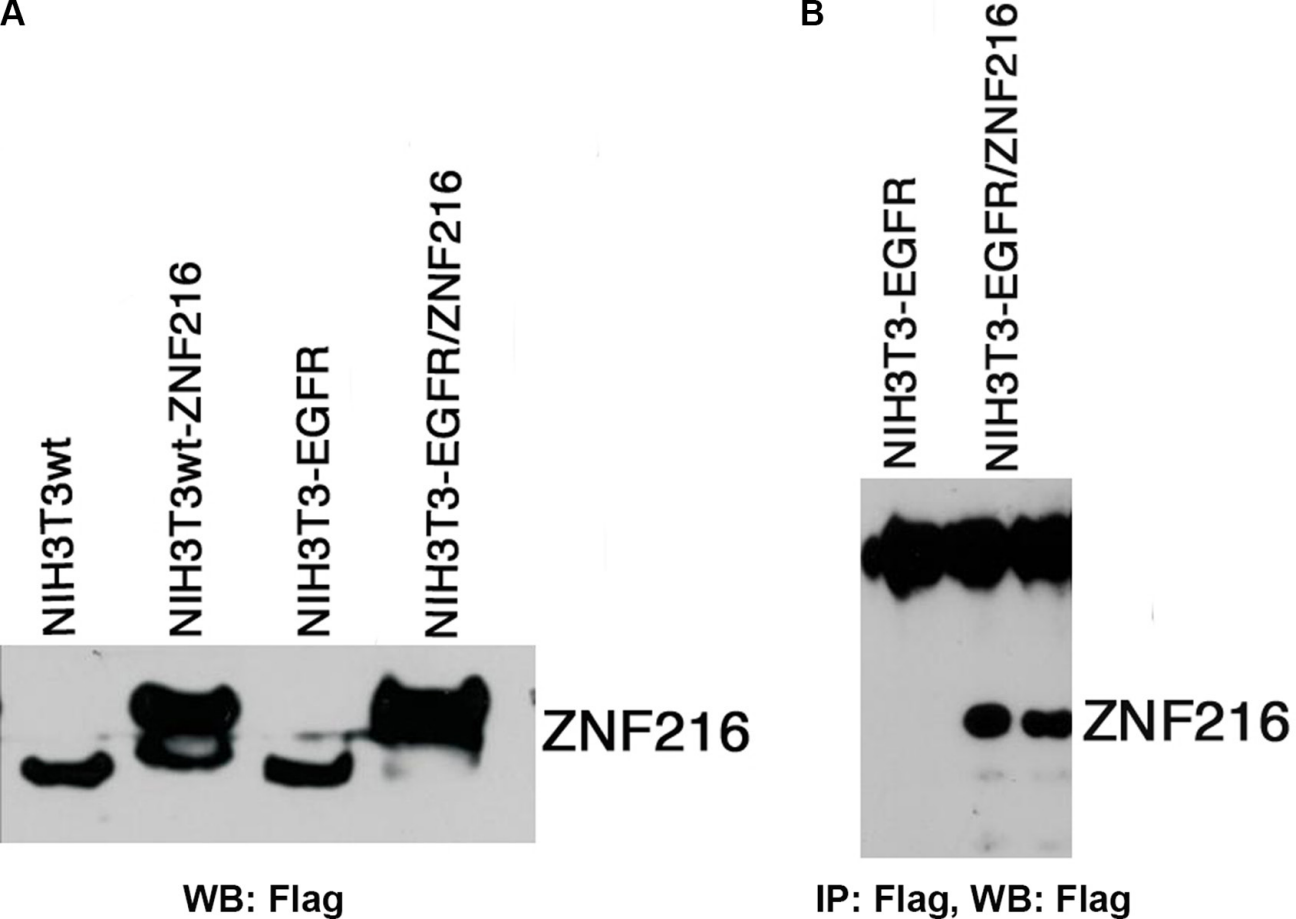
Ectopic expression of ZNF216 protein (**A**) NIH3T3wt cells and NIH3T3-EGFR cells stably expressing EGFR, were transfected with expression vector containing the pF-hZNF216 cDNA (NIH3T3wt-ZNF216, NIH3T3-EGFR/ZNF216) tagged at its N-terminus with a Flag epitope. (**B**) NIH3T3-EGFR and NIH3T3-EGFR/ZNF216 cells were subjected to immunoprecipitation/Western blotting assay using the indicated antibodies. Results are representative of two independent experiments.

In order to investigate the role of ZNF216 overexpression in EGF-induced cellular signal transduction, the pF-hZNF216 construct, containing the Flag epitope, was used to stably transfect the NIH3T3wt cell line and NIH3T3 cells overexpressing the EGFR gene (Figure 3). To ascertain whether the zeocin-resistant clones were able to express ZNF216 protein, Western blotting (Figure 3A) and immunoprecipitation (Figure 3B) analyses with anti-Flag-M2 were performed. As shown high amounts of ZNF216 protein are present in NIH3T3wt and NIH3T3-EGFR transfected with the pF-hZNF216 construct compared with respective parental cell lines.

### Activation of the EGFR is required for EGF-induced ZNF216 expression

**Figure 4:**
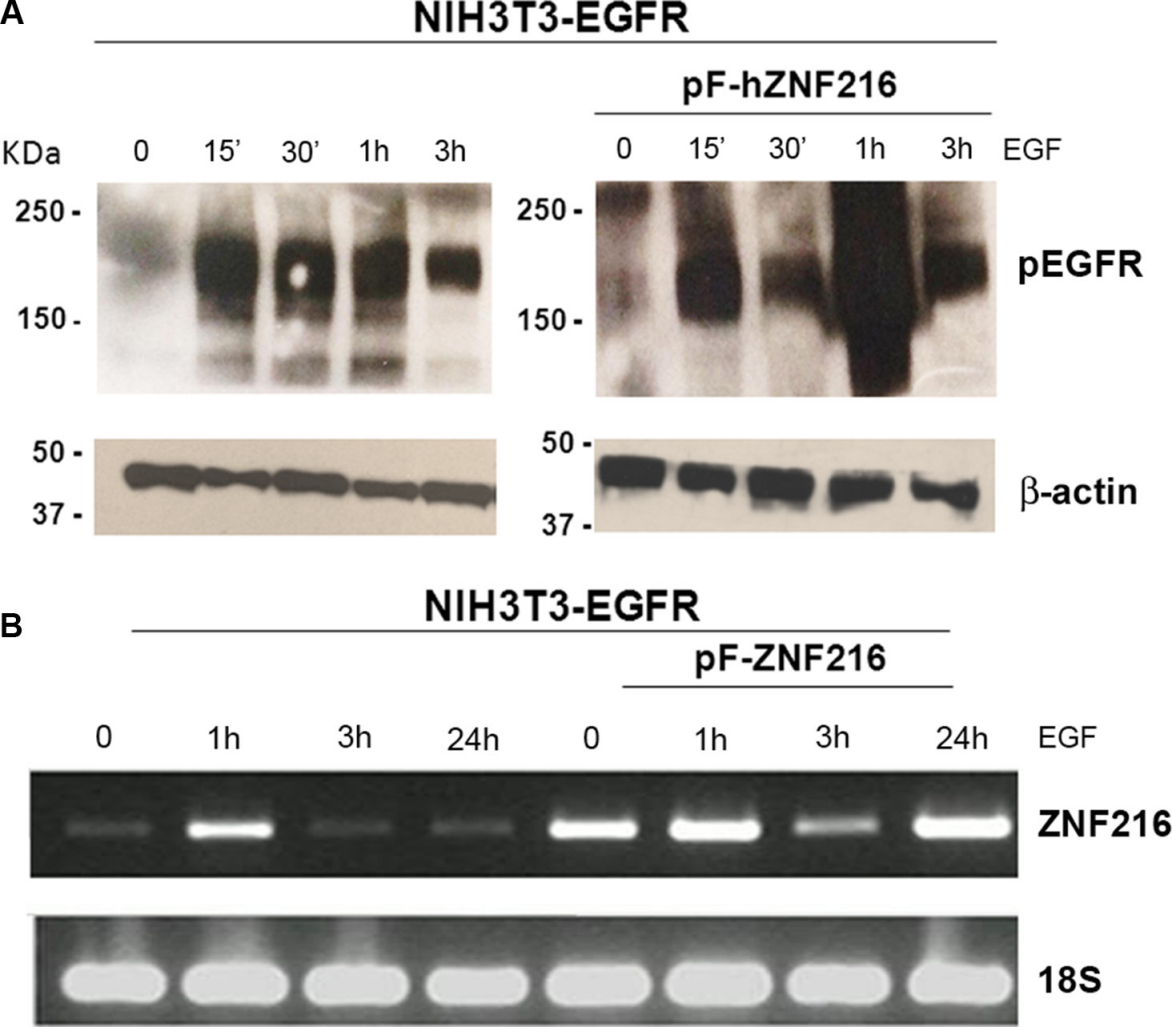
EGF-induced ZNF216 gene expression correlates with EGFR phosphorylation NIH3T3-EGFR and NIH3T3-EGFR/ZNF216 cells, first cultured in medium with 0,2% serum for an overnight and then treated without or with EGF for the time indicated, were analysed by (**A**) Western blotting to evaluate the levels of pEGFR protein and (**B**) by RT-PCR to determine the levels of ZNF216 transcript. β-actin and 18S were used as loading control. Results are representative of two independent experiments.

First, in the attempt to verify whether ZNF216 expression was regulated by EGF treatment, we examined the activation status of the EGFR by Western blotting analysis and the ZNF216 mRNA expression by semiquantitative RT-PCR, both in NIH3T3-EGFR and in NIH3T3-EGFR/ZNF216 cells. A time course study of EGFR phosphorylation in NIH3T3-EGFR cells was carried out showing a fast kinetics of activation at 15 min of treatment compared to the unstimulated cells, remaining stable after 30 min and 1 h and decreasing thereafter (Figure 4A, left). In NIH3T3-EGFR/ZNF216 cells EGFR activation increased at 15 min, slightly decreased at 30 min reached a peak after 1 h of EGF treatment, and then decreased again at 3 h (Figure 4A, right).

A low but detectable level of expression of ZNF216 mRNA was observed in serum starved NIH3T3-EGFR. An immediate early activation of ZNF216 transcription was observed at 1 h after EGF treatment that decreased after 3 h and longer (up to 24 h) (Figure 4B, left). A similar trend was shown in NIH3T3-EGFR/ZNF216 (Figure 4B, right), in according with the EGFR phosphorylation behaviour/activity. However, interestingly, a second peak of ZNF216 mRNA induction was observed after 24 h of EGF treatment.

**Figure 5:**
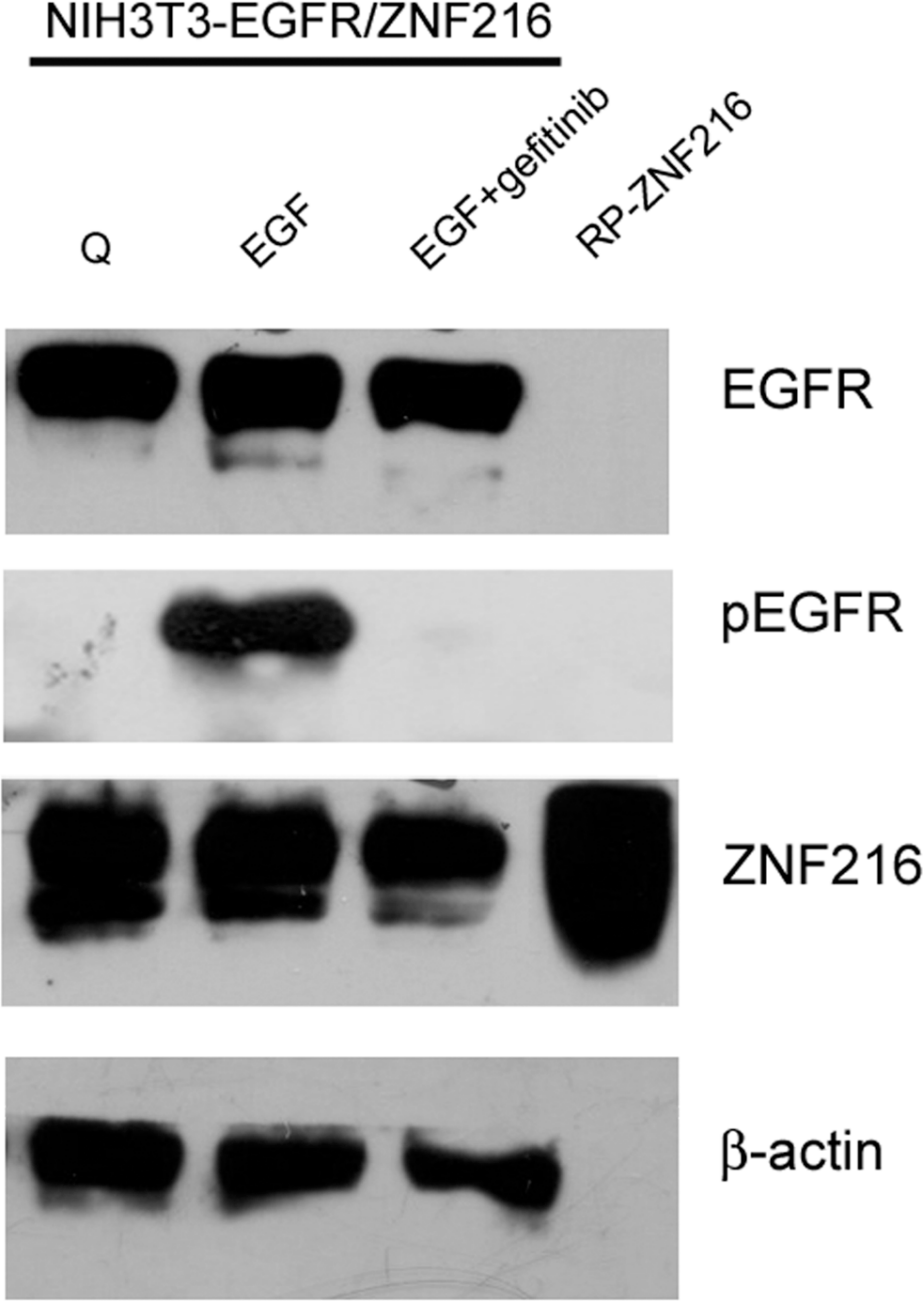
Effect of Gefitinib on EGF-induced ZNF216 protein expression The NIH3T3-EGFR/ZNF216 cells were cultured in medium with 0,2% serum for an overnight, then exposed to EGF alone or in combination with Gefitinib (1 μM) for 24 h and lysed. Whole-cell extracts were analysed by Western blotting using antibody for EGFR, pEGFR and ZNF216. μ-actin immunoreactivity was used as a loading control. RP-ZNF216: Recombinant Protein ZNF216. Results are representative of two independent experiments.

To further examine the involvement of EGFR phosphorylation in the regulation of ZNF216 expression, NIH3T3-EGFR/ZNF216 cells were treated with the EGFR small molecule tyrosine kinase inhibitor Gefitinib. The results obtained clearly indicated that the expression of ZNF216 was no longer increased under co-treatment with EGF and Gefitinib in parallel with the complete abrogation of EGFR phosphorylation (Figure 5), thus suggesting that these events might be coupled. Altogether these results indicate that EGF treatment can result in EGFR activation and consequentially in an increased expression of ZNF216.

### EGF-dependent nuclear translocation of EGFR and ZNF216

**Figure 6:**
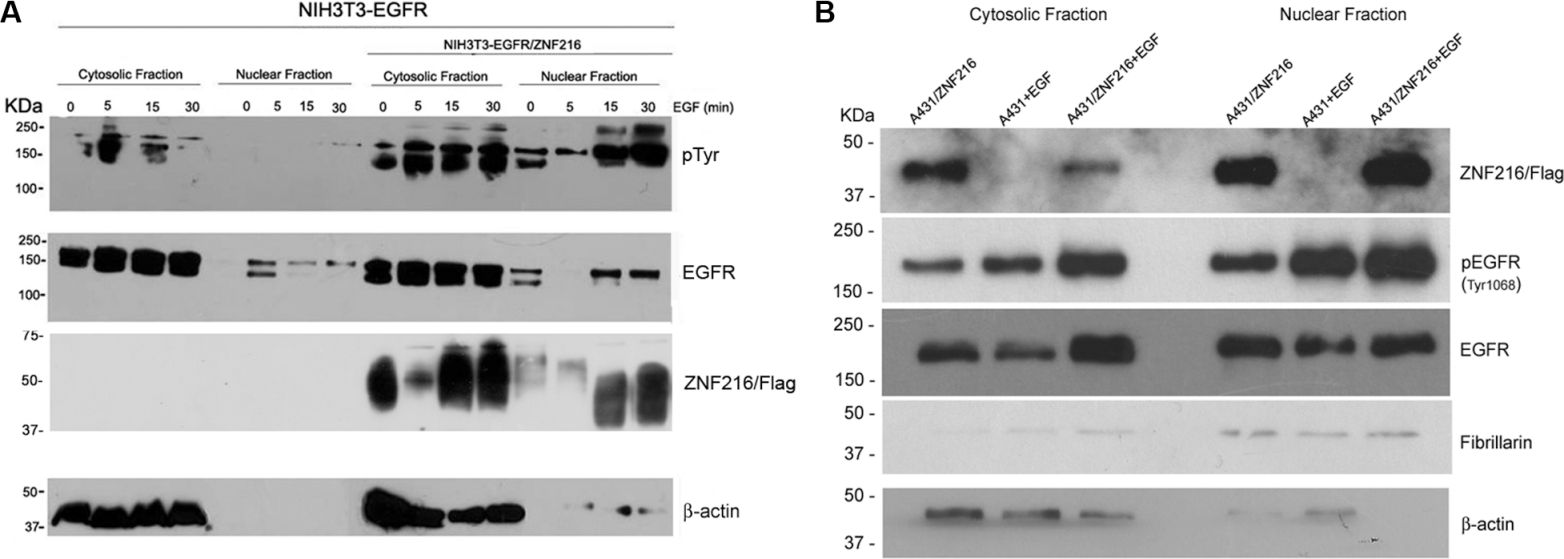
Subcellular localization of activated EGFR in NIH3T3-EGFR and A431 cell lines overexpressing ZNF216 protein after EGF treatment (**A**) NIH3T3-EGFR and NIH3T3-EGFR/ZNF216 cells were cultured in medium with 0,2% serum for an overnight, left unstimulated (0) or stimulated for 5, 15 and 30 min with 50ng/ml EGF and then fractionated into nuclear and cytosolic compartments for Western blotting analysis. The membrane was first probed with anti-phosphotyrosine (p-Tyr, upper panel), stripped and reprobed with anti-EGFR (middle panel) antibody. Western blots, performed by antibody against the cytoplasmic protein β-actin indicated that the extracts are virtually free from cross contamination. Western blotting with the anti-Flag antibody to assess the cellular localization of ZNF216. Results are representative of three independent experiments. (**B**) A431 and A431/ZNF216 cells were treated for 30 min with 50ng/ml EGF and then fractionated into nuclear and cytosolic fractions for Western blotting analysis. Western blots, performed by antibody against the cytoplasmic protein β-actin and against fibrillarin, nuclear protein, indicated that the extracts are virtually free from cross contamination. Results are representative of three independent experiments.

It was reported that the EGFR can be activated and then translocated into the nucleus as a transcriptional activator, and that this nuclear translocation of the EGFR is highly correlated with malignancy [12]. Therefore, in a first series of experiments, to examine whether EGF treatment could influence the intracellular localization of EGFR and ZNF216 in NIH3T3 overexpressing both EGFR and ZNF216 proteins, we carried out a Western blotting analysis on cytosolic and nuclear fractions prepared from cells serum starved for an overnight followed by a 5, 15 and 30 min incubation with EGF (50 ng/ml). In EGF treated NIH3T3-EGFR cells the EGFR appeared mostly and markedly phosphorylated in the cytosolic fraction than in the nuclear fraction (Figure 6A, upper panel). Interestingly, in NIH3T3-EGFR/ZNF216, EGF treatment induced an increase of phosphorylated EGFR in the cytosolic fraction and also in the nuclear fraction, compared to NIH3T3-EGFR cells.

Thus, these results indicate that in the presence of ZNF216 overexpression, phosphorylated EGFR translocates into the nucleus. Moreover, this result suggests that the ligand-activated EGFR did not undergo the endocytosis degradation process, but went through the nuclear pathway to probably induce gene transcription.

It has been previously shown that ZNF216 protein is localized largely in the cytoplasm and to a lesser extent in the nucleus [22]. In an attempt to verify the effect of EGF treatment on ZNF216 cellular localization in NIH3T3-EGFR/ZNF216 we carried out a Western blotting analysis by using an anti-Flag antibody. The staining of ZNF216 was almost intense in the cytosolic fraction of unstimulated cells but strongest after EGF treatment, with a stronger signal after 15 and 30 min of EGF treatment compared to the nuclear fraction of unstimulated cells and after 5 min of EGF treatment (Figure 6A, lower panel). Therefore, these results demonstrate that EGF is able to stimulate increased expression of ZNF216 and its nuclear translocation in a time-dependent manner.

To further confirm the results obtained in the NIH3T3 murine model cell line, we moved our interest on human epidermoid carcinoma cell line A431 that endogenously overexpresses the EGFR. We treated starved A431 cell line and A431 cell line transiently transfected with pF-ZNF216 with EGF for 30 min. Thus, we carried out a Western blotting analysis on cytosolic and nuclear fractions of A431 and A431/ZNF216 cell lines and we proved a similar trend compared to NIH3T3-EGFR and NIH3T3-EGFR/ZNF216. In fact, the EGF treatment increased the EGFR phosphorylation in A431/ZNF216 cells compared to A431 cells, both in the cytosolic fraction and in the nuclear fraction (Figure 6B). Moreover, in the nuclear fraction of A431 cells overexpressing the ZNF216 protein a major amount of phosphorylated EGFR was observed after EGF treatment compared to the cytosolic fraction, according with NIH3T3-EGFR/ZNF216 cell line, suggesting a possible migration of activated EGFR into the nucleus after EGF binding. Interestingly, after EGF treatment, the ZNF216 protein was more expressed in the nucleus than in the cytosol of A431/ZNF216 cell line, validating the hypothesis that ZNF216 protein migrates into the nucleus after EGF treatment along with the activated EGFR (Figure 6B).

**Figure 7:**
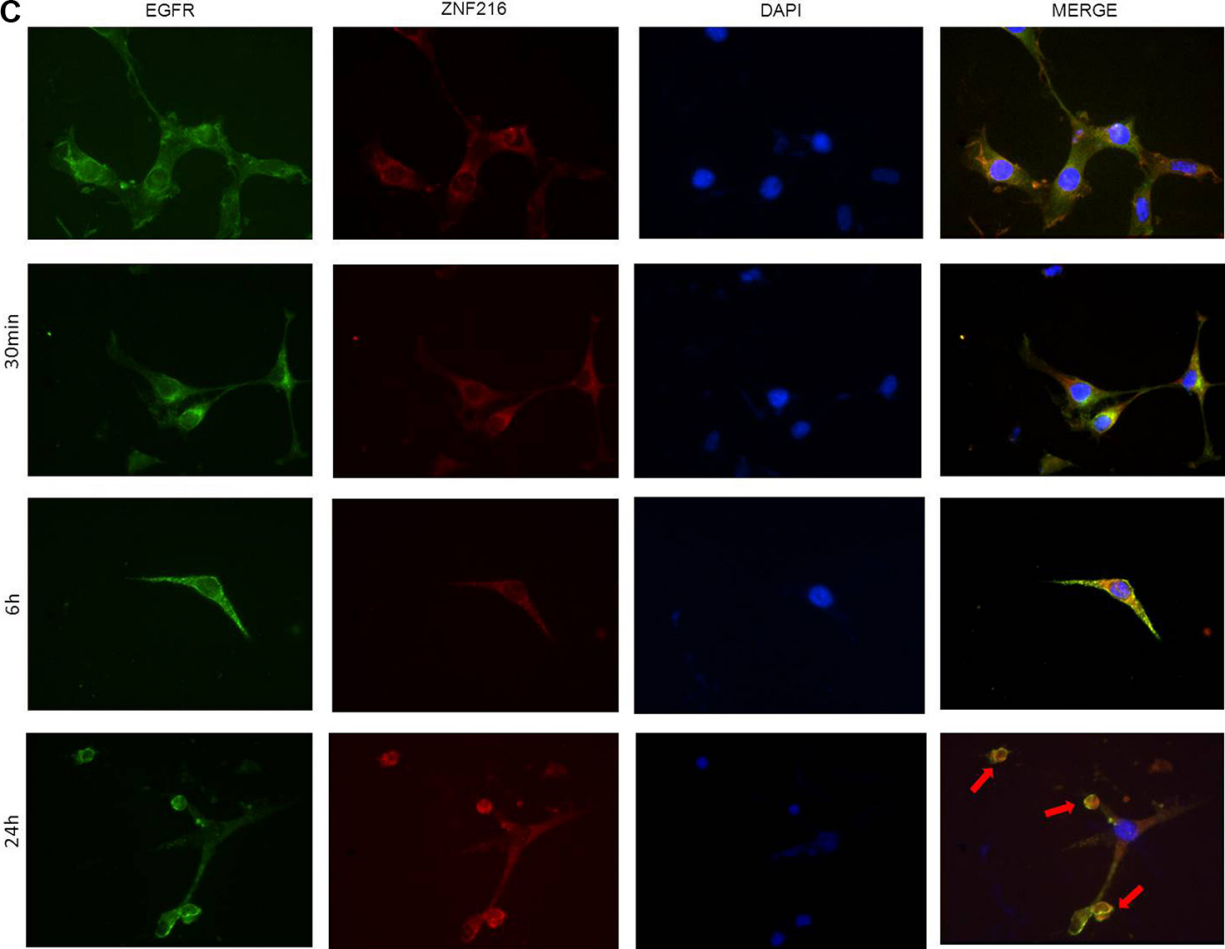
Immunofluorescence localization of EGFR and ZNF216 NIH3T3-EGFR/ZNF216 cells were cultured in medium with 0,2% serum for an overnight, left unstimulated (**C**) or stimulated for 30 min, 6 and 24 h with 50ng/ml EGF. EGFR is visualized as green fluorescence, ZNF216 as red fluorescence and DAPI positive nuclei as blue fluorescence. The merge of the three colors is reported in the right column. Arrows show apoptotic cells. Magnification is 40X. Images are representative of five separate experiments

Finally, the EGFR and ZNF216 subcellular localization was evaluated with an immunofluorescence assay (Figure 7), in the absence and presence of EGF (50 ng/ml, 30 min, 6 and 24 h) by staining with anti-EGFR and anti-Flag antibodies. Before stimulation, EGFR (green) was clearly localized and homogenously distributed to the membrane, while ZNF216 (red) was consistently distributed in the cytoplasm. EGF treatments induced protein redistribution: after 30 min EGFR and ZNF216 co-localized (yellow signals) in the perinuclear region, but after 6 h a significant fraction of ZNF216 was detected in the nucleus. This result supports the hypothesis of a possible cooperation between EGFR and ZNF216 protein, and even though we failed to demonstrate a physical association with co-immunoprecipitation experiments (data not shown) further studies are necessary to clarify this mechanism.

After 24 h, the distribution and localization of EGFR and ZNF216 was similar to the control. The image revealed the presence of apoptotic cells, probably due to a cytotoxic effect of the treatment. After 24 h of treatment with EGF, the morphology of NIH3T3-EGFR/ZNF216 cells showed cell membrane blebbing, pyknotic nuclei with chromatin condensation, and formation of apoptotic bodies.

### Stimulation of EGFR-dependent gene activation by ZNF216 in NIH3T3 cells overexpressing EGFR

**Figure 8:**
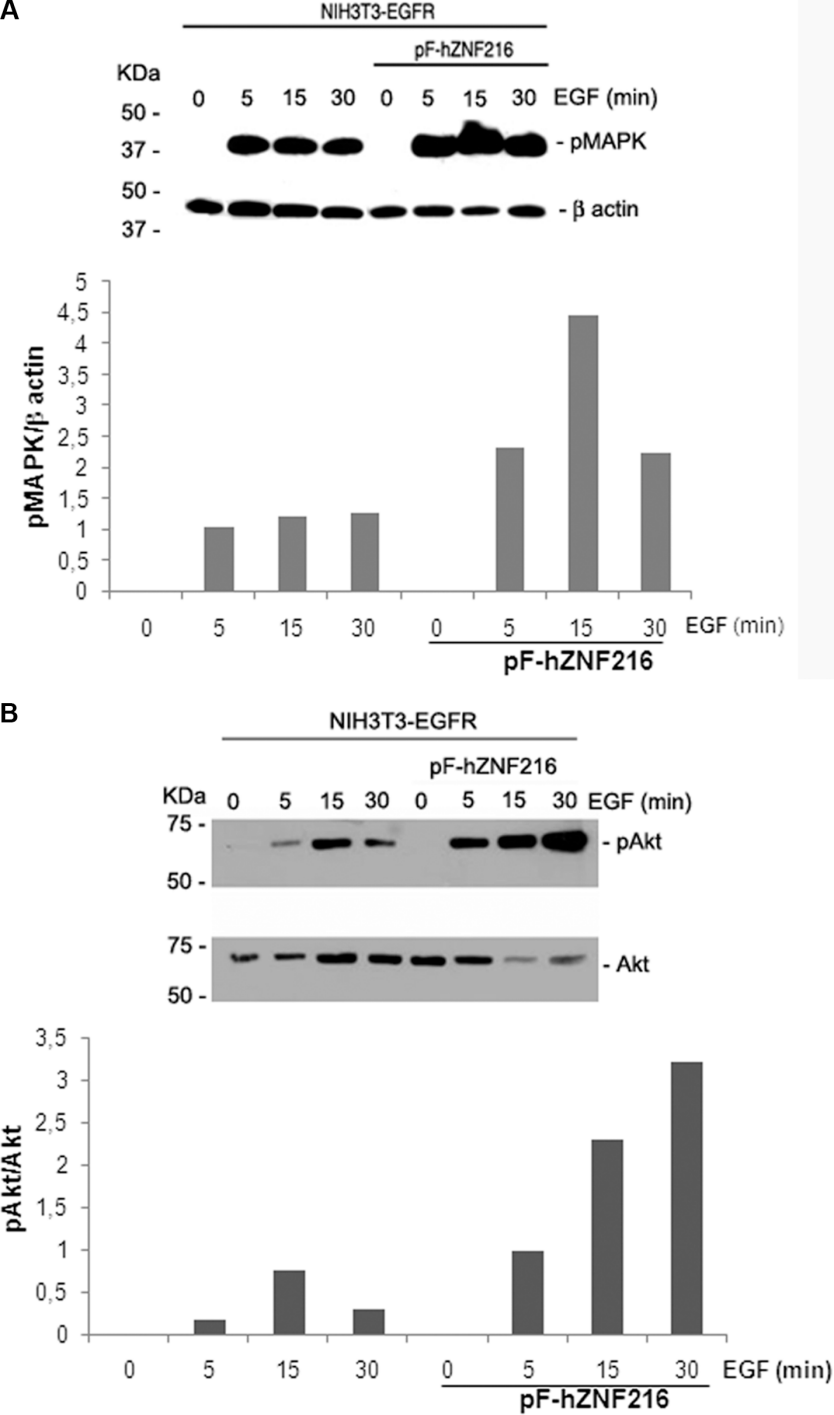
Effect of EGF on MAPK and Akt activation NIH3T3-EGFR and NIH3T3-EGFR/ZNF216 cells were cultured in medium with 0,2% serum for an overnight, left unstimulated and then treated with EGF (50ng/ml) for the times indicated. Proteins lysates were subjected to Western blotting for phosphorylated MAPK and for β-actin (A, upper and lower panel) and for phosphorylated and total Akt (B, upper and lower panel). Reaction products were quantified by computer-assisted densitometry. Results are representative of three independent experiments.

The successive step was to evaluate in which way ZNF216 overexpression could regulate/modulate the signaling cascades triggered by EGFR. Phosphorylation of EGFR by EGF led to the activation of the MAPK and PI3K/Akt pathways that play an important role in cell proliferation and survival. NIH3T3-EGFR and NIH3T3-EGFR/ZNF216 cells were serum starved for an overnight followed by a 5, 15 and 30 min incubation with EGF (50 ng/ml). The results obtained showed an EGF-induced time-dependent activation of MAPK (Figure 8A) and Akt (Figure 8B) in NIH3T3-EGFR/ZNF216. The phosphorylation of Akt in NIH3T3-EGFR/ZNF216 cells was relatively long lasting when compared with the Akt activation in NIH3T3-EGFR cells.

### Biological effects of ZNF216 overexpression

**So far we demonstrated that:**

1. an inverse correlation does exist between EGFR and ZNF216 molecules, both at mRNA and protein levels;
2. EGF increased the expression of ZNF216 both in the cytosolic and in the nuclear fraction in a time-dependent manner;
3. in cells overexpressing ZNF216 protein, EGF increased EGFR phosphorylation in both the cytosolic and the nuclear fraction compared to control cells;
4. a sustained phosphorylation of MAPK and Akt was observed after EGF treatment in ZNF216 overexpressing cells.

Therefore, in the next set of experiments we investigated the biological effects of the increased phosphorylation of EGFR, MAPK and Akt in cells overexpressing the ZNF216 protein.

**Figure 9:**
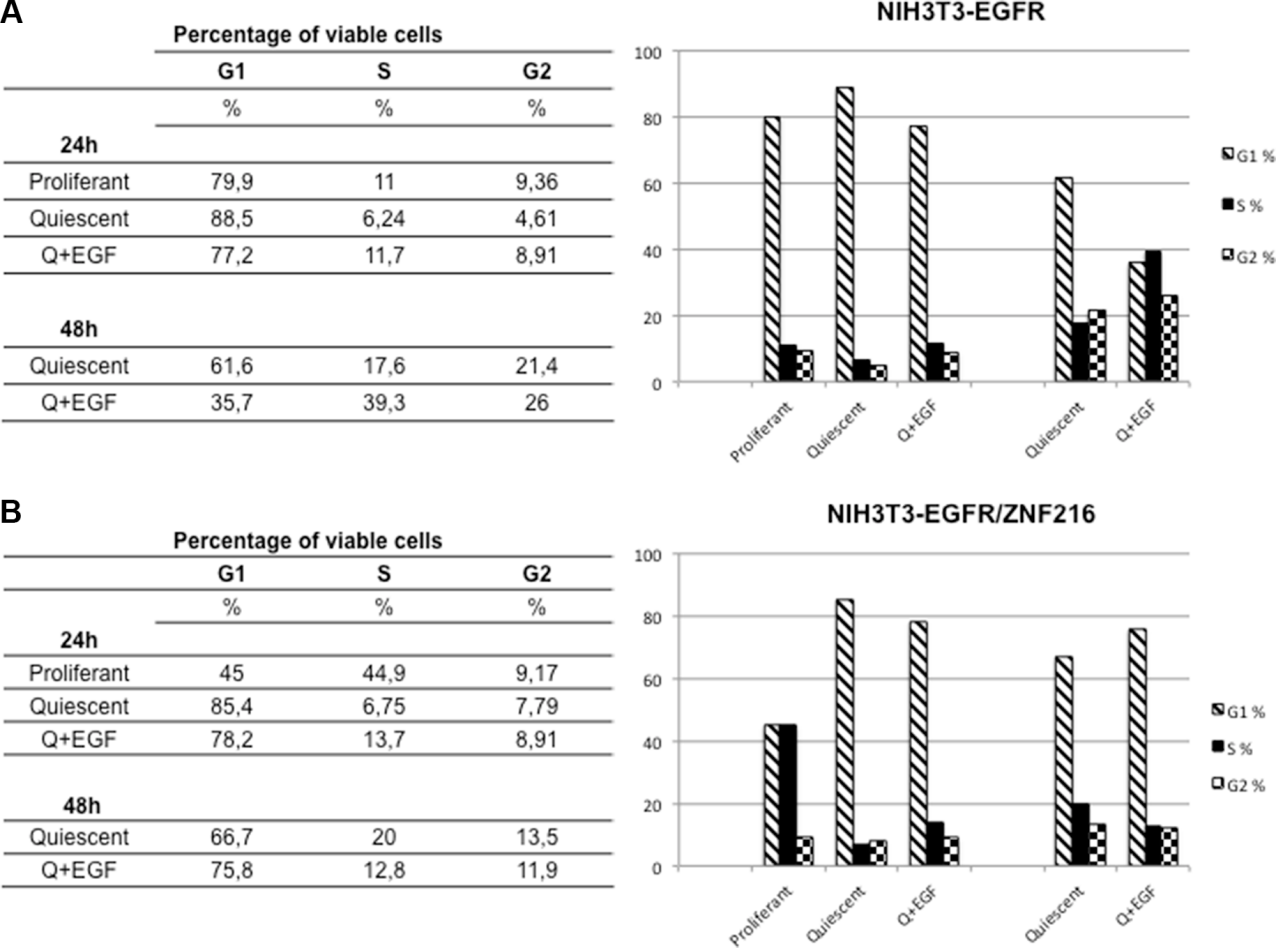
Cell cycle analysis of NIH3T3-EGFR and NIH3T3-EGFR/ZNF216 cells treated with EGF NIH3T3-EGFR (**A**) and NIH3T3-EGFR/ZNF216 (**B**) cells were cultured in medium with 0,2% serum for an overnight, left unstimulated and then treated with EGF (50ng/ml) for the times indicated. Cytofluorimetric analysis was performed in cells that are in the different phases of cell cycle. The data, representative of three independent experiments, were performed in duplicate.

First, cell cycle analysis was performed to evaluate changes in the cell cycle distribution after EGF treatment of ZNF216 overexpressing cells. While NIH3T3-EGFR cells (Figure 9A) continued to progress into S phase after EGF treatment from 24 h (quiescent 6.24%, EGF-treated 11.7%) to 48 h (quiescent 17.6%, EGF-treated 39.3%), NIH3T3-EGFR overexpressing ZNF216 protein (Figure 9B) displayed a progression toward S phase after 24 h of EGF treatment (quiescent 6.75%, EGF-treated 13.7%) but a loss of S phase cells from 20% in quiescent cells to 12.8% after 48 h of EGF-treatment. In parallel, NIH3T3-EGFR/ZNF216 cells accumulate in G_1_ phase more than NIH3T3-EGFR after 48 h of EGF-treatment (75.8% and 35.7%, respectively) (Figure 9).

Therefore, these results showed that EGF treatment caused in NIH3T3 overexpressing ZNF216 protein a decrease in the S-phase population with accumulation of cells in the G_1_ phase of the cell cycle.

### ZNF216-induced apoptosis in NIH3T3-EGFR cells *in vitro*

**Figure 10:**
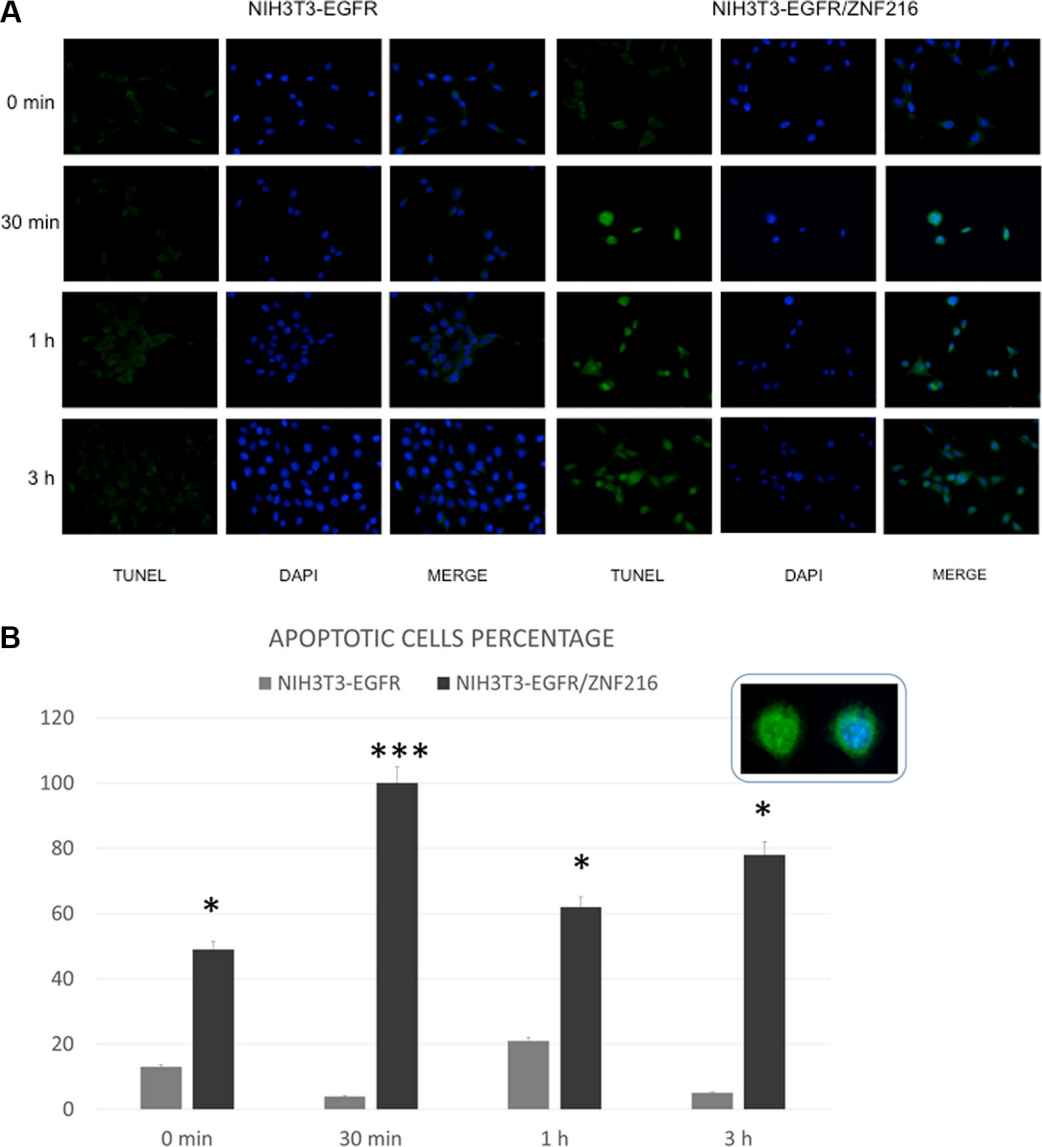
EGF-induced apoptosis in NIH3T3-EGFR overexpressing the ZNF216 protein (**A**) Immunofluorescence labelling of NIH3T3-EGFR and NIH3T3-EGFR/ZNF216 after treatment with 50 ng/ml of EGF for different time intervals (0 min, 30 min, 1 h, 3 h). (**B**) The histogram shows the mean percentage ± S.E. of apoptotic cells detected in NIH3T3-EGFR and NIH3T3-EGFR/ZNF216. The inset shows an apoptotic cell labelled with TUNEL technique (green fluorescence) or double-labelled with TUNEL plus 6-diamino-2-phenylindole (DAPI) (blue fluorescence) to counterstain nucleus. Original magnification: 20x. NIH3TR3-EGFR vs NIH3TR3-EGFR/ZNF216: 0 min p=0.015; 30 min p=3.49×10-9; 1 h p=0.031; 3 h p=0.015.

**Figure 11:**
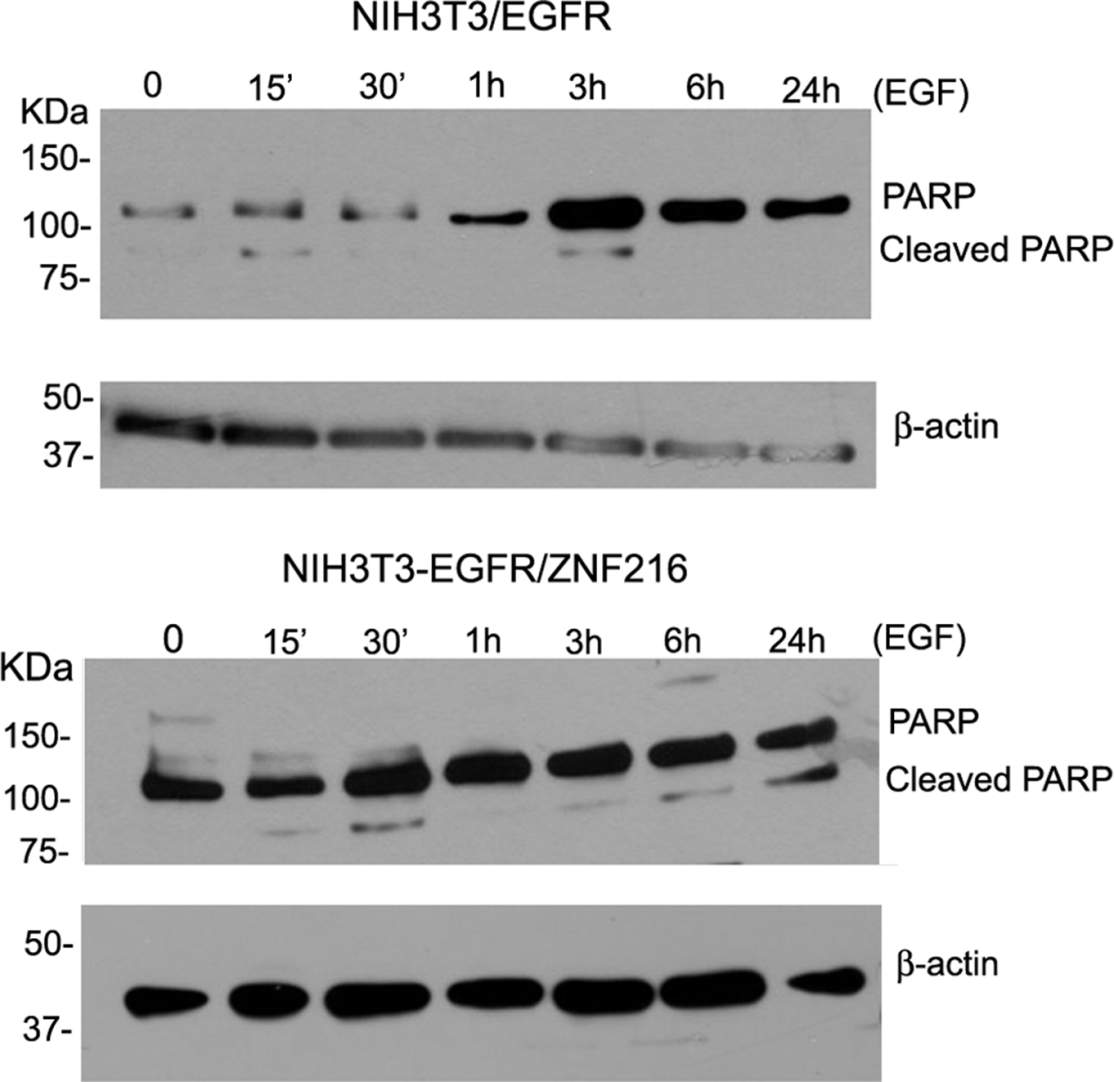
Effect of EGF on cleaved PARP NIH3T3-EGFR and NIH3T3-EGFR/ZNF216 cells were cultured in medium with 0,2% serum for an overnight, left unstimulated and then treated with EGF (50ng/ml) for the times indicated. Cellular proteins were subjected to Western blotting for PARP and β-actin. Results are representative of three independent experiments.

ZNF216 was shown to inhibit Tumor Necrosis Factor (TNF), IL-1 and TLR4-induced NF-κB activation and its overexpression sensitized cells to TNF-induced apoptosis. In order to evaluate possible apoptotic effects of ZNF216, NIH3T3-EGFR cells overexpressing ZNF216 protein were treated with 50 ng/mL EGF up to 24 h and analysed by TUNEL assay. It was interesting to note that in the absence of stimuli no induction of apoptosis was displayed; instead, a significantly increased positivity for the terminal deoxynucleotidyl transferase (TdT)-mediated nick end labeling (TUNEL) was found in NIH3T3-EGFR/ZNF216 samples treated with EGF indicative of apoptotic cell death (Figure 10). Moreover, besides apoptotic nuclei, a number of cells displayed an increased amount of cytoplasm and an overall increased size already after 30 min EGF treatment in consistence with the occurrence of the G_1_ block detected with flow cytometry from 48 h onwards (Figure 9B). To further confirm the apoptotic effect of ZNF216 in NIH3T3-EGFR cells and overexpressing ZNF216, we investigated the status of PARP protein in cells treated with EGF at different time points. The results obtained indicated that PARP cleavage was evident in cells overexpressing ZNF216 protein from 15 min and sustained up 24 h compared to NIH3T3-EGFR cells (Figure 11), confirming previous results obtained.

## DISCUSSION

In this study we demonstrated that the ZNF216 expression is induced by EGF treatment and that it is involved in the regulation of Epidermal Growth Factor Receptor (EGFR) tyrosine kinase signaling and biological outcomes. The EGFR, also known as ErbB1, is one of four members of the ErbBs family of transmembrane growth factor receptor with tyrosine kinase (TK) activity, and it is activated by a number of ligands, including EGF. EGFR controls cell proliferation and survival [39, 40] and its cellular activity is dysregulated in cancer by several mechanisms including gene mutation, gene amplification, and protein overexpression [41, 42]. Binding of EGF to EGFR activates the tyrosine kinase activity of the receptor, resulting in autophosphorylation of tyrosine residues in the carboxy-terminal domain and in activation of downstream signaling cascades that culminate in cell fate decisions [2]. Moreover, after autophosphorylation, EGFR is ubiquitinated and targeted for internalization, even if the ubiquitin-based mechanism has been ruled out as essential for EGFR internalization [43]. However, ubiquitination is also thought to act as a signal for post-endocytotic processing, leading EGFR through the endosomal pathway to either subsequent degradation in the lysosome or escape recycling route to the plasma membrane [44] where receptor functionality is maintained [45].

EGFR is activated, degraded and recycled depending to a complex signaling in which time and context restrictions (e.g. ligand type, cell density) are the leading factors thus, influencing the EGFR-dependent biological effects, such as cells' proliferation, survival, adhesion, differentiation, and migration [18]. Thus, EGFR aberrant activity and its signaling complexity are crucial in the pathogenesis of human cancer. The sophisticated regulations of feedback and feedforward loops that characterize its signaling processes are worth to be extensively studied [46].

ZNF216 is expressed in brain, hearth, colon, spleen, kidney, liver, small intestine, placenta, lung and peripheral blood leukocyte but highly expressed in skeletal muscle [25, 26, 31]. However, no information related to ZNF216 expression compared to EGFR expression in human cancer cell lines was available. Thus, first of all we investigated the endogenous expression of this protein in a panel of five human tumor cell lines differently expressing EGFR. As an initial approach, we tested the mRNA expression by RT-PCR analysis. The results obtained revealed that the ZNF216 mRNA was variably expressed among the cell lines (Figure 1A) with the highest level of expression found in melanoma cell line MDA-MB4-35 weakly expressing EGFR and the lowest levels shown in breast carcinoma cell lines T-47D and HBL-100 moderately expressing EGFR mRNA [32]. The ZNF216 protein levels detected on Western blotting (Figure 1B) were almost consistent with the pattern observed at the mRNA levels. The inverse correlation between endogenous ZNF216 and EGFR level of expression was also observed in prostate cancer cell lines, such as LNCaP, DU145 and PC3 cells.

After these preliminary data, we moved our attention from human cancer cell lines model to a murine not transformed cell line model, to investigate the role of ZNF216 in cancerogenesis, *in vitro*. In particular, we used NIH3T3 cells, which normally express a low number of functional EGF receptors (~3 × 10^3^ receptors per cell) [38] and extensively used to assess the transforming activities of different oncogenes [47]. Our results indicated that EGF treatment induced ZNF216 mRNA and protein expression both in NIH3T3 cells overexpressing EGFR and in NIH3T3-EGFR/ZNF216 cells. In particular, in our study we found an early induction of ZNF216 protein expression after EGF treatment (Figure 6A). Moreover, we showed that ZNF216 induction is dependent from EGFR tyrosine kinase activity since Gefitinib treatment decreased ZNF216 protein expression in EGF-treated NIH3T3-EGFR/ZNF216 cells (Figure 5). Additionally, in our study ZNF216 mRNA expression was sustained for 1 h (Figure 4B), decreased after 3 h but could still be detected after 24 h of EGF only in NIH3T3-EGFR/ZNF216 cells. Signaling pathways activated by EGFR may result in the transcription of a set of “immediate early genes” (IEGs) [48, 49], whose induction does not require protein synthesis and thus involves latent transcriptional activators already present in cells [50, 51]. The proteins encoded by IE genes are transcription factors or signaling pathway regulators which can further affect cellular gene expression profiles and hence promote phenotypic changes, such as those observed in cancer. In the study of Schmahl et al., *Znf216* (Zfand5) has been found to be an immediate early gene (IEG) involved, as a transcription factor, in neonatal survival in mice [23]. The induction of ZNF216 protein expression after EGF treatment for 30 min (Figure 6) is in accordance with previous studies characterizing the ZNF216 as an IEG induced by RTKs, such as PDGFR [23]. In fact, it has been previously observed that EGFR activation induced many of the same genes as PDGFR, even if at a lower level of induction [52]. The transcriptional program induced by growth factor stimulation involved distinct classes of genes: immediate early genes and secondary response genes that are induced later than immediate early genes [53]. Therefore, the induction kinetics of *Znf216* gene in response to EGF treatment gains insight into the possible functional differences of *Znf216*, as an immediate early gene and as a secondary response gene.

Computational analysis allows us to find out some binding sites for the transcription factor Early growth response-1 (*Egr1*) into the *Znf216* gene promoter. Egr1 encodes a zinc finger transcription factor that exemplifies a group of immediate early response genes, since a variety of growth factors, cytokines, rapidly and transiently induce its expression. The induced EGR1 in turn binds to the EGR response element in the promoter regions of growth factors and cytokines and up-regulates these genes. Thus, EGR1 may function as a converging point for many signaling pathways [54]. Among the growth factors, EGF has been shown to strongly induce Egr1 expression through MAPK-ERK pathway [54, 55]. It has been shown that *Znf216* is induced at a transcriptional level by TNF-α or IL-1 in fibroblasts or macrophages [31] suggesting that is a part of a negative feedback loop aimed at limiting pro-inflammatory signaling likely by inhibiting NF-kB activation in response to TNFR or IL-1/TLR signaling in epithelial cells lines [26]. Here we demonstrated that *Znf216* is an EGF-induced gene.

Moreover, our results indicated that EGF stimulation increases the nuclear localization of ZNF216 protein in NIH3T3 cell line transfected with ZNF216 more than in the cytoplasmic fraction, suggesting a possible role played by this protein in the nuclear compartment. Concurrently pEGFR, in according with literature, increases both in the cytoplasm and in the nucleus after EGF stimulation, but this is clearly more evident in NIH3T3-EGFR/ZNF216 than in NIH3T3-EGFR cell line, suggesting a possible role of the ZNF216 protein to act with phosphorylated EGFR in the nucleus after EGF treatment. By immunofluorescence we demonstrated that EGFR and ZNF216 co-localized in the perinuclear region, but after 6 h a significant fraction of ZNF216 was detected in the nucleus. In the context of membrane receptor tyrosine kinases signaling, different zinc finger proteins are involved. Indeed, Enigma is able to bind to the insulin receptor and the zinc finger protein ZPR1 to the EGFR [56].

Sorting of activated EGFR for lysosomal degradation (and therefore attenuation of signaling) or recycling to the plasma membrane (associated with prolonged signaling) is fundamental to the regulation of EGFR signaling. However, alternative fates for activated EGFRs are emerging, including traffic to the nucleus [12, 57]. Nuclear EGFR has two identified functions in the nucleus: 1) as a transcription factor and 2) in the direct phosphorylation of Proliferating Cell Nuclear Antigen (PCNA). As a transcription factor, EGFR has been shown to interact with STAT3 and E2F1 to mediate transcription of cycD1, iNOS, B-myb and Aurora kinase A [58–61, 12, 16]. In our study, the observation that nuclear EGFR is phosphorylated at its autophosphorylation sites indicates that kinase activity of EGFR is present within nucleus and suggests that this kinase activity may be relevant for the function of nuclear EGFR [62]. The fact that expression of ZNF216 is itself under the control of EGFR, suggests that ZNF216 could be involved in the negative feedback regulation of EGFR activation.

Our results indicate that ZNF216 overexpression sensitizes to apoptosis the NIH3T3/EGFR/ZNF216 cells thus antagonizing the effect of activated EGFR-mediated survival in control cells that do not overexpress the ZNF216. The role of ZNF216 in induction of apoptosis has been well established by Huang et al. who demonstrated that although ZNF216 mimics A20 in terms of NF-kB regulation, these two molecules have opposing effects as regulators of cell viability. Indeed, whereas A20 enhances viability of most cell types, overexpression of ZNF216 has been shown to sensitize cells to TNF-induced apoptosis [26] in according with our results. However, this is in disaccording with the over activation of Akt observed in NIH3T3-EGFR/ZNF216 after 30 min of EGF treatment, the same time during which we could observe the highest percentage of apoptotic cells (Figure 10B). In this regard, a report has proven that manipulation of the NF-kB, c-Jun N-terminal kinase, or p38 MAPK signals switches leukemia cells from a proliferative to an apoptotic phenotype leading highly proliferative cells to die rapidly [63]. Moreover, the recruitment of PI3K/Akt and other survival pathways has been demonstrated both in primary cells and leukemia cell lines in response to TRAIL treatment in spite of the induction of apoptosis [64–66]. The fate of the cell would depend on the intensity, kinetics, and synergy of agonistic or antagonistic pathways activated. Thus, we suggest that mechanisms downstream of Akt may interfere with the pro-survival effect induced by EGF.

## MATERIALS AND METHODS

### Cell culture, transfections and stimulation by growth factors

Human epidermoid carcinoma (A431), human breast carcinoma (T-47D, HBL-100, MDA-MB-231), and human melanoma (MDA-MB-435) cells, were maintained in DMEM supplemented with 10% Foetal Bovine Serum (FBS). The NIH3T3 wild type fibroblasts (NIH3T3wt) and NIH3T3 stably overexpressing human EGF receptor (NIH3T3-EGFR) and other receptors of the ErbB family (NIH3T3-ErbB2, NIH3T3-ErbB3, NIH3T3-ErbB4), were grown in DMEM containing 10% Foetal Calf Serum (FCS). These transfectants have previously been characterized and are known to express individual ErbB receptors in the range of 0.6–3 × 10^6^ molecules/cell [67–70].

The three human prostate carcinoma cell lines used in this study, were differentially cultured: the PC3 (androgen-independent) cell line was maintained in DMEM-F12 supplemented with 10% FBS, DU145 cell line (androgen-independent) was grown in DMEM containing 10% FBS and the LNCaP cell line (androgen-dependent) was grown in RPMI with 10% FBS, 10 mM Hepes and 1 mM Sodium pyruvate. Gefitinib (ZD1839) was provided by AstraZeneca (Macclesfield, UK). Stock solutions were prepared in DMSO and then stored at −20°C. For the experiments the cell lines were treated with or without Gefitinib at a concentration of 1 μM, corresponding to serum concentration usually reported in clinical trials.

NIH3T3wt, NIH3T3-EGFR cells were stably transfected with ZNF216 expression plasmid pF-hZNF216 (gift of K. Watanabe) using the Nucleofector^TM^ kit (Lonza, Cologne, Germany) according to the manufacturer's protocol. We used 6 μg of pF-hZNF216 expression vector, which carries a zeocyn resistance gene [22]. Briefly, 1 × 10^6^ cells were pelleted and re-suspended in 100 μl of solution R, and electroporated with 6 μg of DNA. The electroporated cells were re-suspended in 500 μl RPMI transferred into the 6-well plated and incubated for 24 h at 37°C and 5% CO_2_. One day post-transfection, the cells were trypsinized and re-plated in DMEM containing 10% serum and Zeocin (Life Technologies, Carlsband, CA, USA) for selection of stably transfected cells. Drug-resistant colonies were expanded to generate clonal cell lines. Clones were assayed for the expression of ZNF216 by immunoblotting using anti-Flag antibody.

A431 cells (3 × 10^5^) were transient transfected with 4 μg of pF-hZNF216 expression vector using 10 μl of Lipofectamine 2000 (Life Technologies) in Opti-MEM reduced serum medium (GIBCO) for 4 h and then grown in DMEM at 0.2% FBS. After 24 h the cells were stimulated with the addition of 50 ng/ml of EGF (Sigma-Aldrich, St. Louis, MI, USA) for 30 min and then washed once with Phosphate Buffered Saline (PBS) and used for nuclear and cytoplasm fractioning.

### Anti-ZNF216 polyclonal antibody production

On the basis of the ORF coding for hZNF216, two oligonucleotide primers were designed as follows: primer F (carrying a 5′ recognition site for the restriction enzyme BamHI, shown underlined), 5′-CGGGATCCATGGCTCAGGAGACTAACC-3′ (sense) and primer R (carrying a 5′ recognition sites for the restriction enzyme EcoRI, shown underlined), 5′-CGGAATTCTTATATTCTCTGAATTTTTTCAGC-3′ (antisense). PCR was performed to obtain the hZNF216 gene; in addition to the template, pF-hZNF216 kindly provided from Dr. Ken Watanabe, and the primers (F and R), the 50 μl reaction mixture contained 0.2 mM of each dNTP, Pfu DNA polymerase buffer and 2.5 units of Pfu DNA polymerase (Promega, Madison WI, USA). The PCR reaction was subjected to 30 cycles of amplification (60 s at 94°C, 60 s at 55°C and 60 s at 72°C). Both the amplified products and the pGEX-4T-1 expression vector (Life Technologies) were digested with BamHI and EcoRI. The PCR fragment encompassing the complete hZNF216-coding region was ligated into the restricted pGEX-4T-1 vector by using T4 DNA ligase (Boehringer Mannheim, Ingelheim Germany); the resulting plasmid was designated pGEX-hZNF216. An overnight culture of *E. coli* BL21pLys cells transformed with pGEX-hZNF216 was diluted 1:10 and allowed to grow until the OD_600_ reached 0.4. To induce gene transcription, isopropyl β-D-thiogalactoside was added to a final concentration of 1 mM and the incubation was extended for further 5 h. The cells were collected by centrifugation (10,000 × g for 15 min), suspended in PBS and disrupted with cold sonication. The hZNF216 recombinant protein was cleaved by treatment with thrombin, as described by the manufacturer (Life Technologies). The purity of the protein was analysed using SDS/PAGE (12.5% polyacrylamide gels), and proteins were detected with silver staining. PBS containing 100 μg of recombinant hZNF216 was emulsified with Freund's complete adjuvant (1:9, v/v) and subsequently injected into a New Zealand White rabbit. After primary immunization, the animal was given 500 μl of emulsion containing 100 μg of antigen on days 14, 21 and 42; it was then bled 42 days later, and the antiserum was then collected and used for immunoblotting.

### Immunocytofluorescence

To identify the subcellular localization of ZNF216 after EGF treatment, NIH3T3-EGFR cells were stably transfected with ZNF216/FLAG-tagged plasmid (NIH3T3-EGFR/ZNF216). NIH3T3-EGFR/ZNF216 cells were seeded in 24-well plates containing 12 mm round glass coverslips and grown overnight in DMEM 10% FCS. The samples were washed twice and serum-starved for 20 h in DMEM with 0.2% FCS. Samples were then stimulated through the addition of 50 ng/ml of EGF (Sigma-Aldrich) and then incubated at 37°C for the time intervals indicated. After two washes in PBS cells were fixed with freshly prepared 4% paraformaldehyde in PBS (10 min at room temperature), washed in PBS (5 min), and reacted with monoclonal anti-EGFR antibody (1:50) (Dako, Carpenteria CA, USA) and polyclonal anti-Flag antibody (1:200) (Cell Signaling Technology, Beverly, MA, USA) in 150 mM NaCl, 5mM EDTA, 50 mM Tris-HCl, pH 7.4, 0.05% NP-40, 0.25% carragenin Lambda gelatin, 0.02 NaN_3_ (NET gel) for 2 h at room temperature. After several washes, cells were incubated with anti-rabbit IgG Alexa Fluor^®^488 conjugate (diluted 1:500) and anti-mouse IgG Alexa Fluor^®^568 conjugate (diluted 1:500) in NET gel for 45 min at room temperature. After one wash with NET gel and one with PBS, samples were stained (5 min) with PBS 0.5 mg/ml DAPI, then washed in PBS, dried with ethanol (70%, 90%, 100%) and finally mounted in glycerol containing 1,4-diazabicyclo[2.2.2]octane to minimize fading. Negative controls were represented by samples incubated with the secondary antibodies only. Slides were observed with a i50 microscope (Nikon) and images were acquired with a Cool-SNAPcf digital CCD camera (PhotoMetrics, Huntington Beach, CA, USA). Digital acquisition, processing and analysis of fluorescence were performed by Meta Image Series 7.5 (MetaMorph, Metafluor, MetaVue) software obtained from Molecular Devices.

### Immunoprecipitation and Western blotting

The cells with or without growth factor treatment were washed with ice-cold Ca^2+^, Mg^2+^-free PBS and lysed in freshly prepared lysis buffer [2mM Na3VO4, 4 mM sodium pyrophosphate, 10 mM sodium fluoride, 50 mM HEPES pH 7.9, 100 mM NaCl, 10 mM EDTA, 1% Triton X-100, 2 mg/ml leupeptin, 2 mg/ml aprotinin, 1 mM PMSF]. Cell lysate were cleared with centrifugation at 12.000 rpm for 20 min. Protein concentrations were determined using the BCA protein assay (Thermo Fisher Scientific, Waltham, MA USA). For the immunoprecipitation, 500 mg of protein were incubated at 4°C overnight with anti-Flag antibody (1:50) (Cell Signaling Technology). Then, 50 ml of 10% protein-A Sepharose (GE Healthcare Europe, Chalfont St. Giles, UK) were added and incubated at 4°C for 3 h. The precipitates were washed three times with ice-cold immunoprecipitation buffer [10 mM Tris-HCl pH 7.5, 150 mM NaCl, 1 mM EDTA, 1 mM EGTA, 0.2 mM Na3VO4, 0.2 mM PMSF, 1% Triton X-100, 0.5% Nonidet P-40]. All samples were then boiled for 5 min in sample buffer containing 0.5 M Tris-HCl, pH 6.8, 10% glycerol, 2% SDS, 0,1% bromophenol blu, 5% β-mercaptoethanol. Samples were then run on SDS-PAGE. Resolved proteins were electrophoretically transferred to PVDF membrane (Bio-Rad Laboratories, CA, USA). The membranes were incubated in blocking buffer for 1 h and then incubated with antibodies against the following proteins: anti-Flag, anti-phospho-p44/42 MAPK, anti-phospho-Akt, anti-Akt, anti-phospho-EGFR (Tyr1068), anti-PARP (Cell Signaling Technology); anti-Fibrillarin, anti-EGFR (Santa Cruz Biotechnology, CA, USA); anti-PTyr PY20 (Transduction Laboratories, KY, USA); anti-β-actin (Sigma-Aldrich) and anti-ZNF216. Primary antibodies were detected through peroxidase-conjugated relative secondary antibodies. Finally, the immune complexes were visualized using the ECL Western blot detection system (Thermo Fisher Scientific).

### Cytoplasmic and nuclear fractionation

Cytoplasmic and nuclear extracts were prepared from NIH3T3-EGFR, NIH3T3-EGFR/ZNF216 and A431 cells transfected with pF-hZNF216, serum-starved for 20 h in DMEM with 0.2% FCS and treated with 50 ng/ml of EGF (Sigma-Aldrich) for the time intervals indicated. These cells, after washing with cold PBS, were re-suspended in lysis buffer [10 mM HEPES (pH 7.4), 10 mM KCl, 2 mM MgCl_2_, 0.1 mM EDTA] with protease and phosphatase inhibitors, and incubated on ice for 10 min. Nonidet P-40 was added to the cells to a final concentration of 0.5% followed by mixing and centrifugation at 1,000 × g for 5 min at 4°C, and the supernatant was saved as a cytosolic extract. Nuclear extracts were prepared by re-suspending the pellets in 15 ml of 50 mM HEPES (pH 7.8), 50 mM KCl, 300 mM NaCl, 0.1 mM EDTA, 10% glycerol, and protease and phosphatase inhibitors. Proteins were extracted through agitation for 20 min at 4°C, the insoluble fraction was removed by centrifugation at 13,000 × g for 10 min. The supernatant was stored as a nuclear extract. Proteins from nuclear and cytoplasmic fractions were then analysed through Western blotting analysis.

### RNA extraction, reverse transcription-polymerase chain reaction (RT-PCR) and real-time quantitative PCR (qRT-PCR)

Total RNA was extracted from cell lines using Trizol Reagent (Life Technologies) according to the manufacturer's instructions. All RNA samples were examined as to their concentration, purity and integrity based on absorbance ratio at 260/280 nm using NanoDrop 2000 c UV-Vis Spectrophotometer (Thermo Fisher Scientific). Overall sample integrity was confirmed by denaturing formaldehyde agarose gel electrophoresis, showing sharp and intense 18 S and 28 S ribosomal RNA bands with a total absence of smears. Two-step RT-PCR was performed using the QuantiTect Reverse Transcription Kit (QIAGEN GmbH, Hilden, Germany) for cDNA synthesis according to the manufacturer's recommendations and cDNAs were stored at −20°C until use.

Primers for human ZNF216, human EGFR and housekeeping genes 18S, were designed using Gene Works software (Intelli Genetix, Inc., Mountain View, CA, USA) and Allele ID, PREMIER Biosoft International (Corina Way Palo Alto CA, USA). The RT-PCR reactions were performed using 1 ml (of 20 ml) of cDNA template amplified in a total volume of 20 ml, containing 200 mmol/L each of all four deoxynucleoside triphosphates, 2 mmol/L each of specific primers and 1unit of Taq DNA Polymerase (QIAGEN GmbH). Quantitative Real-Time PCR assay was carried out in an Eppendorf Master cycler EP RealPlex (Eppendorf AG, Hamburg, Germany). For all reaction cDNA templates 1 ml was used in a 20 ml Real Time quantitative PCR amplification system of SYBR Green real Master Mix kit (Promega) according to the manufacturer's recommendations. The data obtained have been converted into correct input files, according to the requirements of the software and analyzed using VBA applet and BioGazelle for the gene of interest.

### Cell cycle analysis

The number of cells in each phase of the cycle was evaluated by means of flow cytometry after Propidium Iodide (PI) staining of ethanol-fixed cells. The samples were serum-starved for 20 h in DMEM with 0.2% FCS, treated with 50 ng/ml of EGF (Sigma-Aldrich) and then incubated for the time intervals indicated. The 5 × 10^5^ cell samples were vortexed in 200 ml of PBS and fixed with 500 ml of ice-cold 70% ethanol at 4°C for at least 2 h. The cells were then centrifuged, washed once in PBS, re-suspended in 500 ml PBS and incubated in the dark at room temperature for 30 min in the presence of 100 mg/ml RNAse and 20 mg/ml PI. The PI fluorescence of individual nuclei was measured using FAC Scan (Becton-Dickinson, San Josè, CA, USA). The proportions of cells in the G_0_/G_1_, S and G_2_/M phases of the cell cycle were automatically calculated by the Lysis II analysis software (Becton-Dickinson).

### Immunofluorescent staining of DNA strand breaks (TUNEL)

The TUNEL assay detects single or double DNA strand breaks by means of labelled nucleotides polymerized to free 3′-hydroxyl termini in a reaction catalyzed by Terminal deoxynucleotidyl Transferase (TdT). The NIH3T3-EGFR and NIH3T3-EGFR/ZNF216 cells grown in 24 well plates were serum starved for 20 h in DMEM supplemented with 0.2% FCS. After treatment with 50 ng/ml EGF for 30 min, 1 and 3 h, the cells were fixed with 4% paraformaldehyde. Samples were incubated in a permeabilizing solution (0.1% Triton X-100, 0.1% sodium citrate) for 2 min on ice. Deoxyribonucleic acid strand breaks were identified with an “In situ cell death detection kit” (Boehringer Mannheim, Mannheim, Germany) according to the manufacturer's instructions. Slides were counterstained with DAPI (Vectors Laboratories, Burlingame, CA, USA), mounted in glycerol, and observed with a ZEISS Axioskop light microscope equipped with a CoolSNAP video camera (CoolSNAP; Photometrics, Tucson, AZ, USA) for acquiring digital images. The extent of DNA fragmentation was quantified through direct visual counting of labelled nuclei at 40× magnification. At least three monolayers per sample were examined, and apoptotic cells were scored out of a total of 100 cells. Positive control samples consisted of cells treated with deoxyribonuclease I at 2–5 mg/ml for 10 min at room temperature, while negative control samples were performed omitting TdT in the incubation mixture.

## CONCLUSIONS

The negative correlation observed between EGFR and ZNF216 could imply a functional interplay between these two genes. In this model, the lack or functional loss of ZNF216 should potentiate the survival induced by EGFR. A more thorough understanding of the EGFR signaling pathway will facilitate the future success of clinically used anti-EGFR agents and the development of novel therapies that target the EGFR pathway. Our results on ZNF216 functions could have both basic as well as significant clinical relevance: (a) this molecular regulator may be critical in tumor cell-specific EGFR overexpression, a common occurrence in a variety of epithelial tumors; and (b) ZNF216 could be a novel therapeutic target, up-regulation of which could contribute to the degradation of EGFR protein leading to reduced clonogenic survival of EGFR-addicted cancer cells. In short, the findings paint a picture of two molecules, EGFR and ZNF216, that are linked and provide a signature for the crosstalk between them, offering an additional readout to the potential function of ZNF216. However, the results here shown based on the capability of ZNF216 to inhibit EGFR pro-survival effect could provide a new molecule as a potential target for intervention even if future functional studies aimed at clarifying the mechanism through which this occurs and at analyzing their expression in human cancer compared to paired adjacent normal tissues whom clinical data is available, are clearly warranted. Understanding the dynamics of this receptor, whose signaling is highly regulated in both subcellular localization and kinetics by several extracellular and intracellular processes, will be crucial in identifying potential new targets. Epidemiological and further functional *in vitro* studies are required to establish the reliability of *EGFR* and *Znf216* genes as possible valid molecular targets in the pharmacological strategies aimed at controlling human tumorigenesis.

## Abbreviations

ZNF216: Zinc Finger 216
EGFR: Epidermal Growth Factor Receptor
